# Editorial: Androgen-dependent diseases and their treatment

**DOI:** 10.3389/fendo.2024.1526537

**Published:** 2024-12-10

**Authors:** Marisa Cabeza

**Affiliations:** Departamento de Sistemas Biológicos, Universidad Autónoma Metropolitana-Xochimilco, Mexico City, Mexico

**Keywords:** androgen-dependent diseases, metastatic castration-resistant prostate cancer (mCRPC), polycystic ovary syndrome (PCO), hyperandrogenism, benign prostatic hyperplasia, 11-oxygenated androgens

This compilation of articles addresses androgen-dependent diseases and their therapeutic approaches. Despite significant advancements in treatment options for conditions such as metastatic castration-resistant prostate cancer (CRPC), benign prostatic hyperplasia (BPH), and polycystic ovary syndrome (PCOS) in women, these health issues continue to pose challenges for researchers. It is important to note, however, that there is currently no therapy available that is free of side effects and capable of effectively improving the management of these androgen-dependent diseases.

CRPC results from abnormal interactions and regulation among the molecules involved in androgen signaling. In this context, the study conducted by Jian et al. provides an extensive review of the literature, guiding us into a realm where genes, RNA, proteins, and metabolites associated with CRPC are systematically classified and functionally compared throughout the evolution of this condition. In their work, the authors detail the mutations and aberrant expression of genes that either overexpress or inhibit the signaling function of the androgen receptor, thereby driving the progression of the disease.

The current therapeutic landscape for this disease has been reviewed through the meta-analysis conducted by Chen et al., which aimed to evaluate the efficacy of novel combined clinical treatments as reported in the scientific literature. Their analysis focused on the poly (ADP-ribose) polymerase inhibitor (PARPi) olaparib as a monotherapy and in conjunction with the CYP17 inhibitor, abiraterone, a steroid derivative. Additionally, the study examined the impact of combining the androgen receptor antagonist apalutamide with abiraterone, specifically assessing its correlation with radiographic progression-free tumor survival. By reviewing 741 bibliographic references, the researchers concluded that patients receiving olaparib as a standalone treatment exhibited longer survival times than those treated with the combination of apalutamide and abiraterone. Although significant differences between the two treatment combined modalities were not established, it is noteworthy that the combination of olaparib, apalutamide, and abiraterone needed to be demonstrated in the analysis.

Hyperandrogenism developed in some women is recognized as a primary contributor to polycystic ovary syndrome (PCOS), where the expression of androgen receptors in various tissues—including the brain, adipose tissue, and liver—plays a significant role in the presentation of symptoms indicative of this syndrome. In the Research Topic, Wang et al. provide a comprehensive review of the literature on this subject, demonstrating that elevated androgen levels result from excessive ovarian androgen production and the synthesis of 11-oxygenated androgens in the adrenal cortex. This phenomenon is associated with specific polymorphisms in the CYP11A1 and CYP17 gene promoters. Moreover, the identification of androstenedione as a diagnostic marker for PCOS with hyperandrogenism is a noteworthy outcome of this research.

Benign prostatic hyperplasia (BPH) is another androgen-dependent disease that continues to worry physicians. It is a disease common in about 50% of older men, so its frequency could burden public health. In clinical practice, it is necessary to know the number of people who can perceive the symptoms of this disease so that they can seek treatment. This condition can be improved surgically by the internal scrapping of the prostate. However, men greatly feared this surgical method. Therefore, pharmacological therapy with 5α-reductase inhibitors, such as dutasteride or finasteride, is an alternative to avoid surgery. However, this pharmacological therapy could be unattainable for poor people. In this regard, Alzahrani et al. conducted a study in Saudi Arabia to determine the prevalence of BPH to improve healthcare planning, resource allocation, public awareness, early detection, intervention, research, and treatment of regional variations. In this study, they conclude that men in this country generally show sufficient knowledge about the symptoms caused by BPH, so most of them go to the doctor. However, the data show that there is a knowledge gap regarding certain risk factors for the development of this disease, such as obesity and heart disease.

This matter may captivate the attention of researchers focused on androgen-dependent diseases, urologists, and endocrinologists due to its comprehensive and up-to-date overview of studies conducted in this domain.

